# The Nine Pillars of Integrated Care, Revisited

**DOI:** 10.5334/ijic.11118

**Published:** 2026-06-09

**Authors:** Niamh Lennox-Chhugani

**Affiliations:** 1 International Foundation for Integrated Care, United Kingdom

**Keywords:** Pillars, integrated care, validation, knowledge

When the International Foundation for Integrated Care (IFIC) first published the Nine Pillars of Integrated Care in 2020, the world was in the early months of the COVID-19 pandemic. The framework, conceived as a Call to Action to accelerate the global shift to integrated care, articulated nine conditions that any health and care system needs in place for integration to sustain and scale [1]. The Pillars rapidly found resonance with policymakers, system leaders and practitioners across IFIC’s international network, and became a reference point for IFIC when organising conferences, structuring evaluations and informing system reform.

Six years on, three realities had made revision not just advisable but necessary. The Nine Pillars were never formally tested or validated; the language was tied to a specific moment in time and limited the framework’s generalisability across contexts and over time; and the wider field of integrated care had continued to develop, adding to the already extensive body of frameworks, models and taxonomies that have emerged over the past fifteen years [2]. The revision and validation work undertaken between 2024 and 2025 set out to address each of these limitations.

## How revision adds to the field of knowledge and practice

There are three contributions that the revised Nine Pillars of Integrated care make to the field of knowledge and practice in integrated care.

Firstly, the Nine Pillars have been used by IFIC as a meta-framework — a higher-order construct that synthesises and organises other frameworks rather than competing with them [3]. In implementation science, frameworks identify and organise key concepts without specifying causal relationships between them [4]; meta-frameworks aggregate and integrate across them. To stand as the structured lens through which integrated care approaches can be compared, assessed and strengthened, the meta-framework needed to be demonstrably consistent with the wider evidence base.

Secondly, a 2024 review conducted by IFIC’s research team with consortium partners under the EU-funded LAUREL project identified 69 integrated care frameworks meeting defined inclusion criteria [5]. These had been developed and applied by researchers, policymakers and implementers across diverse settings. To remain useful, the Nine Pillars needed to demonstrate that they mapped meaningfully against this wider field, and that any gaps revealed through such mapping were addressed.

And finally, there was the question of accessibility. While the Nine Pillars had been adopted readily by experts within IFIC’s network, the language remained dense for non-expert audiences. The policymakers, frontline practitioners and community partners whose engagement is essential to the framework’s practical value needed descriptions that were easier to apply and adapt.

## The methodology in brief

The 14-month revision and validation methodology proceeded in three connected stages.

The first stage was a rapid literature review [6] using the PRISMA 2020 reporting guideline [7] to identify integrated care frameworks across health and social science disciplines. The 69 frameworks identified at the abstract screening stage were narrowed to 26 through full-text screening. These 26 frameworks were then mapped in two ways: first against the system levels (micro, meso and macro) at which they operated, and second against the content of the Nine Pillars (2020).

The system-level mapping confirmed that, while a meta-framework needs to operate across micro, meso and macro levels, many of the reviewed frameworks were not designed to do so: 14 of the 26 frameworks were applicable at all three levels, with a further six applicable across all three but with at least one level only partially addressed. The remainder were targeted at one or two levels. This finding reinforced the value of a meta-framework that could organise these more specialised approaches rather than replace them.

The content mapping yielded several actionable findings. *People as partners in care* was represented in all 26 frameworks and *Workforce capacity and capability* in 20. By contrast, *Aligned payment systems* appeared in only 13 frameworks. Within the workforce Pillar, reviewed frameworks consistently emphasised interprofessional, interdisciplinary or multidisciplinary teamwork as primary mechanisms of integration — a dimension referenced only obliquely in the original Pillar through the language of “blurring of roles”. Crucially, eight frameworks included content related to service design that had no direct counterpart in the Nine Pillars, raising the question of whether a service design Pillar should be added.

The second stage applied a modified Delphi method. Fifty international experts in integrated care, drawn from the Scientific Committee of IFIC’s annual International Conference on Integrated Care, were invited to participate. Of these, 36 expressed interest, 29 completed Round 1, 25 completed Round 2, and 22 completed the final Round 3. Consensus was operationalised at ≥75% agreement, drawing on established methodological guidance [8].

In Round 1, panellists reviewed proposed descriptions and suggested wording changes. Their feedback prompted a substantive structural change: each Pillar would be described in general terms and then, additionally, from the perspectives of three groups: people and communities, care providers, and system-level decision-makers. Rounds 2 and 3 used scoring on progressively narrowed scales to test consensus on the revised descriptions. By Round 3, no Pillar attracted less than 86% consensus, with several reaching 95–100%.

Where dissent persisted, it was substantively recorded. Dissent was centred around the relationship between population health and integrated care, the bias of certain descriptions towards universal coverage models, and the framing of descriptions as ideal “what good looks like” statements. These remain legitimate areas for ongoing development.

The third stage tested the descriptions for accessibility. As part of an introductory course commissioned for non-expert policymakers and care providers, the IFIC team gathered feedback on understandability. This prompted a further refinement: aligning the Pillars to the WHY, WHAT and HOW of integrated care, providing a clearer organising logic for users approaching the framework for the first time.

## What has changed

The revised Nine Pillars retain the essential structure of the 2020 framework while addressing the gaps identified through the process. A new Pillar, *Service delivery for coordinated and integrated care*, was introduced to address the gap revealed through the framework review. The Pillar previously labelled *Resilient communities and new alliances* was integrated into other Pillars, particularly *People and communities as partners in care*. The labels themselves have been tightened: *Shared values and vision* became *Purpose*; *Workforce capacity and capability* became *Teamwork*; *Transparency of progress, results and impact* became *Learning*. Each new label is intended to function as shorthand alongside more detailed descriptions, balancing breadth with specificity. The descriptions themselves have been rewritten in generalisable language, with accounts from the perspective of practitioners, communities and decision-makers giving different groups entry points appropriate to their roles.

This structure, prompted directly by the Delphi process, is a meaningful gain. Each Pillar is now articulated through a general description and three additional accounts: from the point of view of people and communities, of care providers, and of system-level decision-makers. The change makes more visible the fact that integrated care is not a single set of conditions experienced uniformly by everyone in a system, but a set of conditions whose meaning and impact shifts depending on where in the system one stands.

## How the Nine Pillars will continue to evolve

The revised Nine Pillars [9] are not a fixed text but a starting point for further development.

First, IFIC will test the revised Pillars with community groups and people with lived experience over the next 24 months. The feedback gathered through this work will continue to refine the language and framing, ensuring the Pillars remain accessible beyond expert audiences.

Second, IFIC will host a repository for community-based development of the Nine Pillars. Multiple versions are likely to emerge — adapted to specific social, political, historical and structural contexts. Rather than treating these as deviations from a single official text, IFIC will curate them as legitimate iterations that demonstrate the meta-framework’s adaptive use in practice.

Third, ongoing iteration will address areas of remaining dissent. The financing Pillar’s framing, where panellists identified an over-emphasis on state-funded universal coverage, will benefit from the contribution of practitioners working in social insurance and privately financed systems. The relationship between integrated care and the wider determinants of population health, and the inter-dependencies between the Pillars themselves, will also continue to be explored through case studies and practice-sharing.

A maturity self-assessment tool, designed to be adaptable to local context, is also under development. This responds to a thoughtful critique offered during the Delphi process: that descriptions framed as ideal end-states risk feeling unattainable and unmeasurable. The maturity tool will provide practitioners with a means of locating themselves on a journey towards integration, supporting concrete conversations about delivery and progress.

## A framework that earns its place

The revision and validation of the Nine Pillars of Integrated Care marks a transition. From a framework articulated in the urgency of a global pandemic, the Pillars have been re-grounded in the wider evidence base of integrated care, tested through the considered judgement of an international expert panel, and opened to refinement through engagement with non-expert audiences. The result is a more validated, more generalisable and more accessible meta-framework, one that retains its strategic role within IFIC’s mission while providing researchers, policymakers and practitioners with a stronger conceptual tool. As the field continues to evolve, so too will the Pillars. That is by design.

## References

[1] Lewis L, Ehrenberg N. Realising the true value of integrated care: Beyond COVID-19. Oxford: International Foundation for Integrated Care; 2020.

[2] Amelung VE, Stein V, Suter E, Goodwin N, Nolte E, Balicer R, editors. Handbook Integrated Care. 2nd ed. Cham: Springer; 2021. DOI: 10.1007/978-3-030-69262-9

[3] Foroughi Z, Ebrahimi P, Aryankhesal A, Maleki M, Yazdani S. Toward a theory-led meta-framework for implementing health system resilience analysis studies: a systematic review and critical interpretive synthesis. BMC Public Health. 2022;22(1):287. DOI: 10.1186/s12889-022-12496-3 35151309 PMC8840319

[4] Brady SS, Brubaker L, Fok CS, Gahagan S, Lewis CE, Lewis J, et al. Development of conceptual models to guide public health research, practice, and policy: synthesizing traditional and contemporary paradigms. Health Promot Pract. 2020;21(4):510–524. DOI: 10.1177/1524839919890869 31910039 PMC7869957

[5] Lennox-Chhugani N, Gangas P, Aldasoro E, Alvarez A, Gil Salmerón A, Linnane D. D1.1 Desktop Review of Literature. LAUREL Project: Actionable policies for adopting high-quality integrated long-term care; 2024. Available from: https://laurelproject.eu/wp-content/uploads/2025/10/Laurel_D1.1_DesktopReviewLiterature.pdf

[6] Tricco AC, Antony J, Zarin W, Strifler L, Ghassemi M, Ivory J, et al. A scoping review of rapid review methods. BMC Med. 2015;13(1):224. DOI: 10.1186/s12916-015-0465-6 26377409 PMC4574114

[7] Page MJ, McKenzie JE, Bossuyt PM, Boutron I, Hoffmann TC, Mulrow CD, et al. The PRISMA 2020 statement: an updated guideline for reporting systematic reviews. BMJ. 2021;372:n71. DOI: 10.1136/bmj.n71 PMC8005924 33782057

[8] Diamond IR, Grant RC, Feldman BM, Pencharz PB, Ling SC, Moore AM, et al. Defining consensus: a systematic review recommends methodologic criteria for reporting of Delphi studies. J Clin Epidemiol. 2014;67(4):401–409. DOI: 10.1016/j.jclinepi.2013.12.002 24581294

[9] International Foundation for Integrated Care. Nine Pillars of Integrated Care. https://integratedcarefoundation.org/ific-9-pillars-of-integrated-care (Accessed 22 May 2026).

